# Effect of Mass Proportion of Municipal Solid Waste Incinerator Bottom Ash Layer to Municipal Solid Waste Layer on the Cu and Zn Discharge from Landfill

**DOI:** 10.1155/2016/9687879

**Published:** 2016-12-01

**Authors:** Qingna Kong, Jun Yao, Zhanhong Qiu, Dongsheng Shen

**Affiliations:** ^1^Center of Marine and Ecological Environment Protection, Taizhou University, Linhai 317000, China; ^2^Department of Environmental Engineering, Zhejiang University, Hangzhou 310029, China; ^3^Zhejiang Provincial Key Laboratory of Solid Waste Treatment and Recycling, Zhejiang Gongshang University, Hangzhou 310018, China

## Abstract

Municipal solid waste incinerator (MSWI) bottom ash is often used as the protection layer for the geomembrane and intermediate layer in the landfill. In this study, three sets of simulated landfills with different mass proportion of MSWI bottom ash layer to municipal solid waste (MSW) layer were operated. Cu and Zn concentrations in the leachates and MSW were monitored to investigate the effect of MSWI bottom ash layer on the Cu and Zn discharge from the landfill. The results showed that the Zn discharge was dependent on the mass proportion of MSWI bottom ash layer. The pH of landfill was not notably increased when the mass proportion of MSWI bottom ash layer to MSW layer was 1 : 9, resulting in the enhancement of the Zn discharge. However, Zn discharge was mitigated when the mass proportion was 2 : 8, as the pH of landfill was notably promoted. The discharge of Cu was not dependent on the mass proportion, due to the great affinity of Cu to organic matter. Moreover, Cu and Zn contents of the sub-MSW layer increased due to the MSWI bottom ash layer. Therefore, the MSWI bottom ash layer can increase the potential environmental threat of the landfill.

## 1. Introduction

Due to the primary advantages of hygienic control, volume reduction, mass reduction, and energy recovery, incineration has become an attractive way for the municipal solid waste (MSW) treatment in China [1]. Till 2014, there were a total 166 municipal solid waste incinerators (MSWI) with a treatment capacity of 158,488 t d^−1^ [2]. However, incineration is not a final waste treatment process. Large volume of residue, such as the MSWI bottom ash and fly ash, is produced as a result of the incineration [3]. In 2008, Chinese government enacted the “Standard for Pollution Control on the Landfill Site of Municipal Solid Waste” (GB 16889-2008) [4]. In Section 6.1 of the standard, MSWI bottom ash is allowed to be disposed in MSW landfill sites without any pretreatment. In several countries or areas, such as Japan and Taiwan, MSWI bottom ash is also allowed to be disposed in the MSW landfill sites [5, 6]. It is often used as the protection layer to keep the geomembrane from damage, intermediate layer, and leachate drainage layer instead of natural minerals in the landfill [7].

However, MSWI bottom ash contains high level of heavy metals [8]. The MSWI bottom ash layer will raise the heavy metals contents of the landfill, which can increase the potential threat if the nonlinear behavior and sudden releasing of Cu and Zn occurred [9–11]. Several studies have been done to investigate the effect of the disposal of MSWI bottom ash on the heavy metal release from landfill. For example, Lo and Liao [6] reported that the codisposal of MSWI bottom ash with MSW did not increase the metal release, based on their one year's observation. Inanc et al. [5] also pointed out that the metal leaching was not enhanced by the codisposal. It could be due to the high acid neutralizing capacity (ANC) of the MSWI bottom ash. The ANC of the MSWI bottom ash can neutralize the acidic condition and increase the pH, which help to immobilize the heavy metals. But the case can be distinctive in China. The organic matter level of MSW of China was higher than that of other countries, due to the large proportion of the kitchen waste. The organic matter can be degraded into organic acid, forming acidic condition. In some cases, the pH of landfill drops below 5.0 during the acid formation phase [12]. Such an acidic environment can greatly facilitate the mobility of heavy metals. In fact, the landfill of China has already been connected with the serious heavy metal pollution. It is still questionable whether the MSWI bottom ash layer will exacerbate the heavy metal pollution in such an acidic condition. Due to the high ANC of MSWI bottom ash, the pH of the landfill may be changed when different mass proportions of MSWI bottom ash layer are disposed. As a result, the release of heavy metals from the landfill can change. Therefore, it is necessary to evaluate the subsequent environmental impact when different mass proportions of MSWI bottom ash layer are disposed in landfill. To our knowledge to date there are few studies on this theme. Among the heavy metals, Cu and Zn are of particular concern as their contents in the MSWI bottom ash and MSW are relatively high [8, 9, 13]. Besides, they are reported to have a high toxicity to the surrounding ecosystem [14]. They were thus selected for the discussion herein.

In this study, three simulated landfills, namely, conventional MSW landfill (R1), landfill with the mass proportion of MSWI bottom ash layer to MSW layer of 1 : 9 (R2), and landfill with the mass proportion of MSWI bottom ash layer to MSW layer of 2 : 8 (R2), were set up. The proportion 2 : 8 was chosen because it was close to the practical mass proportion of MSWI bottom ash and MSW in Zhejiang province, China. The proportion 1 : 9 was chosen to evaluate the environmental impact when only a part of MSWI bottom ash was disposed in landfill. The effects of the MSWI bottom ash layer on the discharge of Cu and Zn as well as the Cu and Zn contents in MSW were discussed. The result of this study can provide scientific reference for the heavy metal pollution control when the MSWI bottom ash was used as the protection layer, intermediate layer, and leachate drainage layer in landfill.

## 2. Materials and Methods

### 2.1. Experimental Set-Up

**Figure 1:**
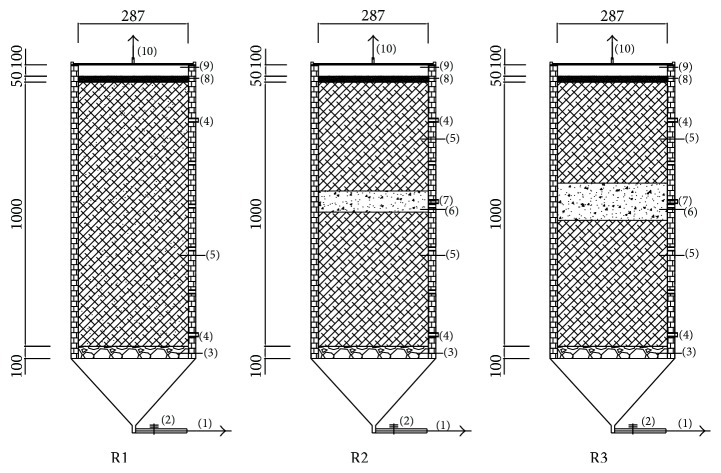
Schematic of simulated landfill systems. (1) Leachate outlet; (2) valve; (3) gravel layer; (4) MSW sampling port; (5) MSW layer; (6) MSWI bottom ash layer; (7) MSWI bottom ash sampling port; (8) sandy layer; (9) headspace; (10) vent port.

Three sets of simulated landfill reactors, including R1, R2, and R3, were set up. Each reactor was 287 mm in diameter and 1000 mm in height, with a working volume of 65 L. Each reactor was equipped with five ports: the outlet port at the top lid was used for exporting gas, and the three ports on the side were used for the sampling of the MSWI bottom ash and MSW, while the remaining bottom one was used for leachate drainage and sampling. MSWI bottom ash was disposed at the middle of R2 and R3. A 100 mm thick layer of gravel, which was chemical inert to the leachate, was placed at the bottom of each reactor to simulate the leachate collection system and to prevent clogging of the leachate withdrawal outlets. Another 100 mm thick layer of gravel with the nominal size from 10 mm to 40 mm was placed at the top of each reactor to simulate cover soil and to ensure the well distribution of the tap water added to the simulated landfills. The schematic diagram of the whole experimental set-up is shown in Figure 1.

### 2.2. MSWI Bottom Ash and MSW

Fresh MSWI bottom ash was taken from the Green Energy MSWI plant in Zhejiang province, East China. The plant consisted of three parallel stoker incinerators with a MSW treatment capacity of 650 t d^−1^. MSWI bottom ash had been through water quenching and magnetic separation before being sampled. MSWI bottom ash was stored in the lab for 15 days before being disposed.

MSW used in this experiment was collected from the Kaixuan transport station of Hangzhou, Zhejiang, China. In order to get a representative sample of solid waste, MSW was collected continuously at different time in the day. Larger particles of the collected MSW were all shredded into 20 mm approximately. Then, they were manually homogenized by shovel as thoroughly as possible prior to loading to the simulated landfills. The main components of the MSW were determined according to the Chinese standard GB/T 19095-2003.

### 2.3. Operation of Simulated Landfills

For R1, 50 kg of MSW was loaded and compacted by a shovel and a sledgehammer. For R2, firstly 22.5 kg of MSW was loaded and compacted. Then, 5 kg of MSWI bottom ash was loaded. At last, another 22.5 kg of MSW was loaded and compacted. For R3, firstly 20 kg of MSW was loaded and compacted. Then, 10 kg of MSWI bottom ash was loaded. At last, another 20 kg of MSW was loaded and compacted. The moisture content of the MSW was adjusted to 75% by adding 21.4, 19.3, and 17.1 L tap water to R1, R2, and R3, respectively. The tap water was added continuously by using the peristaltic pump [15]. Leachate was collected and stored in the sealed glass tank before being sampled and the storage time of the leachate was no longer than 1 day.

### 2.4. Analyses

The contents of individual elements in the MSWI bottom ash were analyzed by ICP-OES after the sample was digested according to the method described by Yamasaki [16]. The moisture content was determined by ASTM D2216. The bulk density was determined by ASTM C29. The pH was determined for the suspension after 24 h equilibration period with liquid-to-solid ratio of 5 : 1. The loss on ignition (LOI) was determined by the Chinese standard GB7876-87. The high acid neutralization capacity of MSWI bottom ash was determined by the method of Johnson et al. [17]. The Cu and Zn contents of the loaded MSW were analyzed by ICP-OES after MSW was digested according to the method described by Yamasaki [16].

Leachate samples were collected weekly from leachate outlet ports (~100 mL). MSW was sampled biweekly from the sample ports at the side of simulated landfills. The collected leachate and MSW were monitored to track the migration of Cu and Zn in the landfills.

Leachate samples were analyzed for pH, dissolved organic carbon (DOC), and Cu and Zn concentrations. MSW samples were analyzed for Cu and Zn contents. All these analyses were performed in accordance with “Standard Methods for the Examination of Water and Wastewater” [18]. Metal analyses were performed using atomic absorption spectrophotometer. Prior to the analysis, each sample was digested with aqua regia according to the standard method [16]. Analyses of metal concentration in MSW and leachate were carried out in triplicate to ensure the validity of the results. Besides, the fractionation of Cu in the leachate was determined according to the size charge fractionation (SCF) procedure suggested by Driscoll [19], which has been adopted to analyze the fractionation of metals in the landfill leachate [20]. By the procedure, the leachate was filtrated through a 0.45 *μ*m screen filter to obtain the fraction particulate and colloidal matter >0.45 *μ*m. The filtered leachate was then passed through a sulphonic acid cation exchange resin. Cu presented as labile complexes and free cation was adsorbed onto the cation exchange resin. Cu remaining in the leachate was the fraction nonlabile complex.

## 3. Results

### 3.1. Main Characteristic of MSWI Bottom Ash and MSW

The main characteristics of MSWI bottom ash used in this study are presented in Table 1. The pH is high (11.17), which is probably due to the high contents of the alkaline hydroxides and minerals of Ca, Al, Fe, and K, such as CaCO_3_, Al_2_O_3_, and Fe_2_O_3_. The contents of Cu and Zn are 314.6 and 1922.0 mg kg^−1^, respectively, which are within the range of the previous reports [8]. The Cu and Zn contents of MSWI bottom ash used in this study are lower than those of other countries, such as Switzerland (4000 mg kg^−1^ for Cu, 3500 mg kg^−1^ for Zn) and Japan (2818 mg kg^−1^ for Cu, 4229 mg kg^−1^ for Zn) [21, 22].

**Figure 2:**
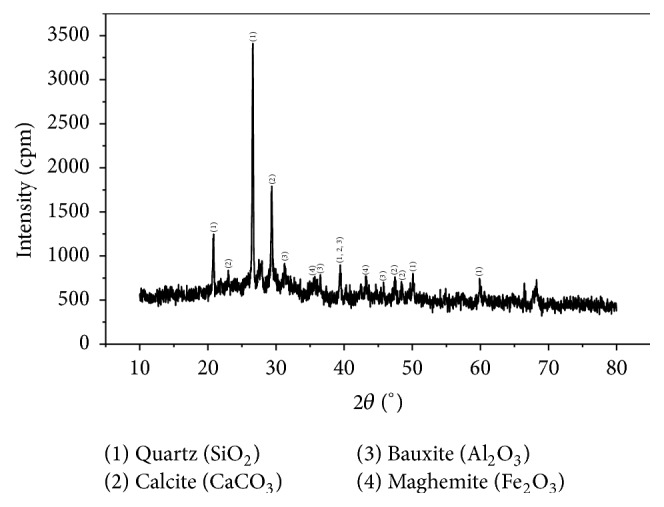
XRD pattern of the MSWI bottom ash.

The X-ray diffractogram of the MSWI bottom ash is shown in Figure 2. The principal minerals identified are quartz (SiO_2_), calcite (CaCO_3_), maghemite (Fe_2_O_3_), and bauxite (Al_2_O_3_). These minerals are the acid consuming substance, indicating the high ANC of MSWI bottom ash, as illustrated in Table 1. The high ANC of MSWI bottom ash may increase the pH of the leachate, if MSWI bottom ash is disposed in the landfill.

The components as well as Cu and Zn contents of MSW are presented in Table 2. It shows that the food waste is the main component, which results in the high level of organic matter in the landfill. The contents of Cu are relatively high in the plastic, timber, and ceramic fraction. The contents of Zn are generally higher than that of Cu. The highest content of Zn is observed in fraction of food waste. These results are consistent with the result of Long [12].

According to the mass and metal content of the MSW and MSWI bottom ash in R1, R2, and R3, it is calculated that there are totally 6464.4 mg of Cu and 57467.5 mg of Zn in R1, 7390.9 mg of Cu and 61330.7 mg of Zn in R2, and 8317.5 mg of Cu and 65193.0 mg of Zn in R3. MSWI bottom ash layer significantly increases the Cu and Zn contents of the simulated landfill.

### 3.2. Variation of the Leachate Characteristic

**Figure 3:**
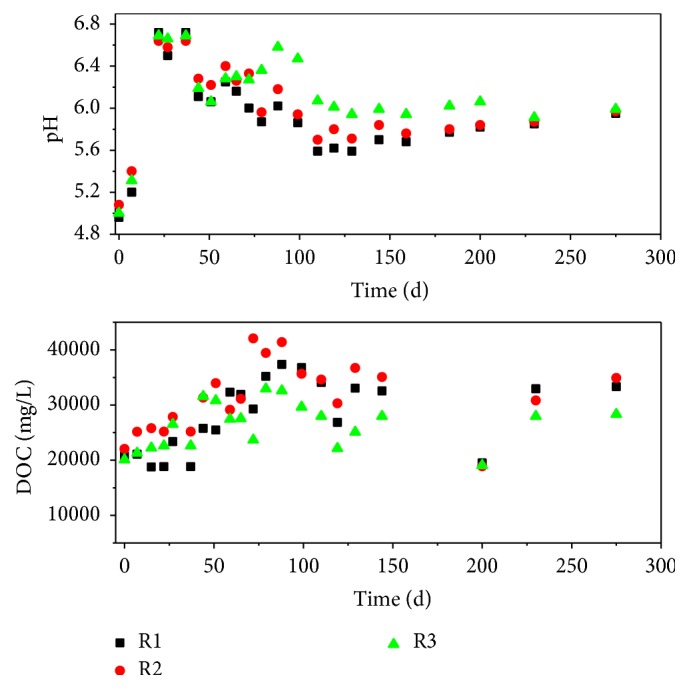
Variation of pH and DOC in the leachate of R1, R2, and R3.

The variation of the pH and DOC of the leachate is shown in Figure 3. The initial pH is low, which is about 5.0 for the three leachates. It shows a fast increase from day 0 to day 37, as the organic acid is consumed by the residual oxygen in the landfill. From day 37 to day 129, the pH shows a reduction, due to the degradation of the MSW into organic acid. The pH kept relatively steady after day 129. From day 44 to day 159, the pH of R2 was slightly higher than that of R1. After day 159, the leachate pH of R1, R2, and R3 was highly close. For R3, the leachate pH was notably higher than that of R1 after day 59. It suggests that the MSWI bottom ash layer is unable to neutralize the acidic condition of the simulated landfill when the mass proportion is 10%. However, the case is different when the mass proportion of the MSWI bottom ash layer is 20%. Besides, a high level of DOC was observed for the three leachates, which exceeded 15000 mg L^−1^ during the study period. The DOC of the leachate experienced an increase from the start to day 80, which might be due to the degradation of MSW. After day 80, the DOC decreased, indicating the degradation of MSW had slowed down. The average concentration of DOC of the leachate of R1, R2, and R3 is 27995.7 mg L^−1^, 31265.1 mg L^−1^, and 26183.7 mg L^−1^, respectively. DOC level of R3 leachate is generally lower than that of R1 and R2.

### 3.3. Discharge of Cu and Zn from Landfill

#### 3.3.1. Cu

**Figure 4:**
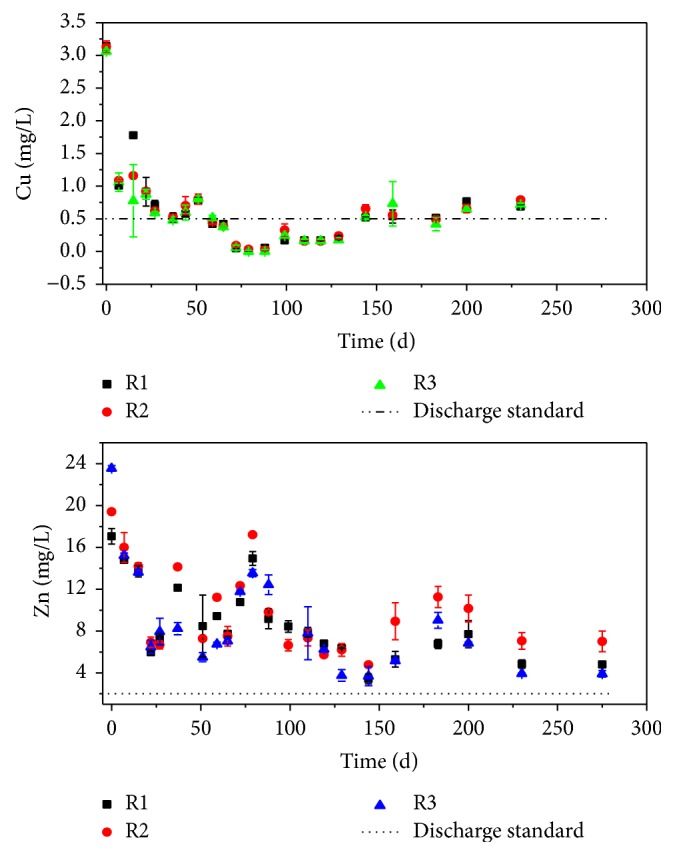
Concentrations of Cu and Zn in the leachate of R1, R2, and R3.

The variation of Cu concentrations in the leachate of R1, R2, and R3 is shown in Figure 4. During the first 150 days, Cu shows in all three experiments a negative trend, which is generally consistent with the previous reports [9, 22]. The initial concentration of Cu is relatively high, which is about 3.1 mg L^−1^ for the three leachates. It is thought that most of the unstable Cu in MSW and MSWI bottom ash is apt to leach out at the beginning of the operation. The concentration of Cu decreased to 0.06 (R1), 0.04 (R2), and 0.01 (R3) mg L^−1^ after day 88. It seems that the leaching and immobilizing of Cu have reached a balance, leading to the low discharge of Cu. However, the concentration of Cu shows a slight rise afterwards, which comes to about 0.7 mg L^−1^ for the three leachates at the end of the study. It may be resulting from the degradation of MSW, releasing a part of Cu bound to the organic matter in MSW [9]. The concentration of Cu in the leachate of the three landfills exceeds the Discharge Standard for Waste Water of China (GB 8987-1996, Grade II: Cu ≦ 0.5 mg L^−1^ for threshold) [23] before day 59 and after day 144. Therefore, it is necessary to monitor the discharge of Cu over a long time period.

**Figure 5:**
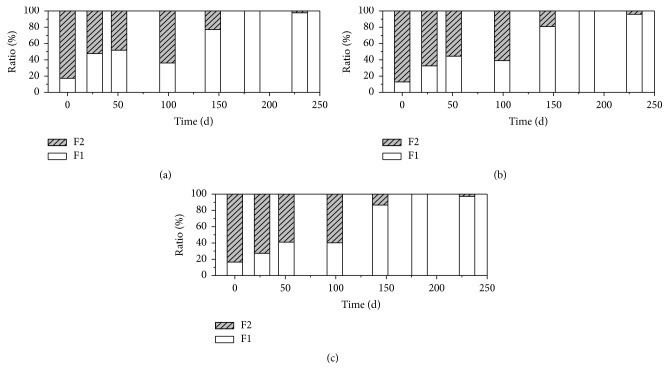
Fractionation of Cu in leachate of R1, R2, and R3 (data are shown as percentage relative to total concentration). (a): R1; (b): R2; (c): R3. F1: particulate and colloidal matter >0.45 *μ*m; F2: nonlabile complex.

In most cases (days 1, 7, 22, 27, 37, 44, 51, 65, 72, 79, 88, 110, 119, 129, 159, and 183), no significant difference of the Cu concentration is observed among the three leachates (*p* < 0.05, shown in Table S1 in Supplementary Material available online at http://dx.doi.org/10.1155/2016/9687879), although the pH and DOC of the leachates vary within a large range (Figure 3). According to the simulate result of Visual MINTEQ, the saturated Cu concentration of leachate should be above 10 mg L^−1^. The Cu concentration in this study is far below the saturated concentration. Therefore, the similar Cu concentration in the three leachates can not be attributed to the fact that the leachate is saturated with Cu. As is well known, Cu has a high affinity for organic ligands. Several researches have found that the leaching of Cu was controlled by organic ligands [24, 25]. The similar leaching behavior of Cu is probably due to the high level of organic ligands in the landfills. This can also be proven by the fractionation of Cu in the leachates (Figure 5), which shows that Cu is almost presented as the particulate and colloidal matter >0.45 *μ*m and nonlabile complex. It means that most of Cu in the leachate is bounded to the organic matter [20]. According to the Cu concentration and the volume of the leachates, the total discharge of Cu from R1, R2, and R3 is calculated (Table S2), which is 12.69 mg, 12.33 mg, and 13.00 mg, respectively. It suggests that the MSWI bottom ash layer may not affect the Cu discharge from the landfill.

#### 3.3.2. Zn

The concentration of Zn in the leachates shows a decreasing trend within the first 144 days. From day 144 to day 200, the Zn concentration shows an increased trend, which could be due to the degradation of MSW. After that, a decreasing trend is observed. The initial concentration of Zn in the leachates follows the sequence of R1 < R2 < R3, which is 17.1, 19.4, and 23.6 mg L^−1^, respectively. This result corresponds to the mass proportion of the MSWI bottom ash layer in the three simulated landfills, indicating that the MSWI bottom ash layer can increase the leachable Zn of the landfill.

In most cases (days 1, 22, 37, 59, 72, 79, 144, 159, 183, 200, 230, and 275), the Zn concentrations of R2 leachate are significantly higher than that of R1. While on days 7, 15, 27, 37, 51, 59, 65, 79, 110, 119, 129, 144, 159, 200, 230, and 275, the Zn concentrations in R3 leachate are significantly lower or at the same level compared to that of R1 (*p* < 0.05, shown in Table S3). For the entire duration of the investigation, the average concentration of Zn in the leachates follows the sequence of R1 < R3 < R2, which is 8.94 mg L^−1^, 9.03 mg L^−1^, and 10.05 mg L^−1^, respectively. These results are not proportional to the mass proportion of the MSWI bottom ash in the simulated landfills. For R2, the MSWI bottom ash layer (mass proportion of 10%) increases the Zn content of the landfill, while the pH is not notably promoted (Figure 3). Thus, Zn leaching is enhanced. For R3, MSWI bottom ash layer (mass proportion of 20%) increases the pH remarkably. As the solubility of Zn in the leachate decreases as pH increases, the migration of Zn is restricted [26]. Therefore, the discharge of Zn from R3 is not greatly increased, although R3 has the highest leachable Zn among the simulated landfills. According to the Zn concentration and the volume of the leachates, the total discharge of Zn from R1, R2, and R3 was calculated (Table S4). A total of 121.99 mg, 145.22 mg, and 111.45 mg of Zn were discharged from R1, R2, and R3, respectively. This result shows that the discharge of Zn from the landfill is dependent on the mass proportion of the MSWI bottom ash layer. When the mass proportion of MSWI bottom ash layer is not high enough to neutralize the acidic condition, the discharge of Zn will be notably enhanced. On the contrary, when the mass proportion of MSWI bottom ash layer is high enough to neutralize the acidic condition, the discharge of Zn can be mitigated.

The Zn concentration of the three leachates exceeds the Discharge Standard for Waste Water of China (GB 8987-1996, Grade II: Zn ≦ 1.0 mg L^−1^ for threshold) during the study period, which is consistent with the result of Johansen and Carlson [27]. The MSWI bottom ash layer with mass proportion of 10% further increases the environmental threat.

### 3.4. Variation of Cu and Zn in MSW with Time

#### 3.4.1. Cu

**Figure 6:**
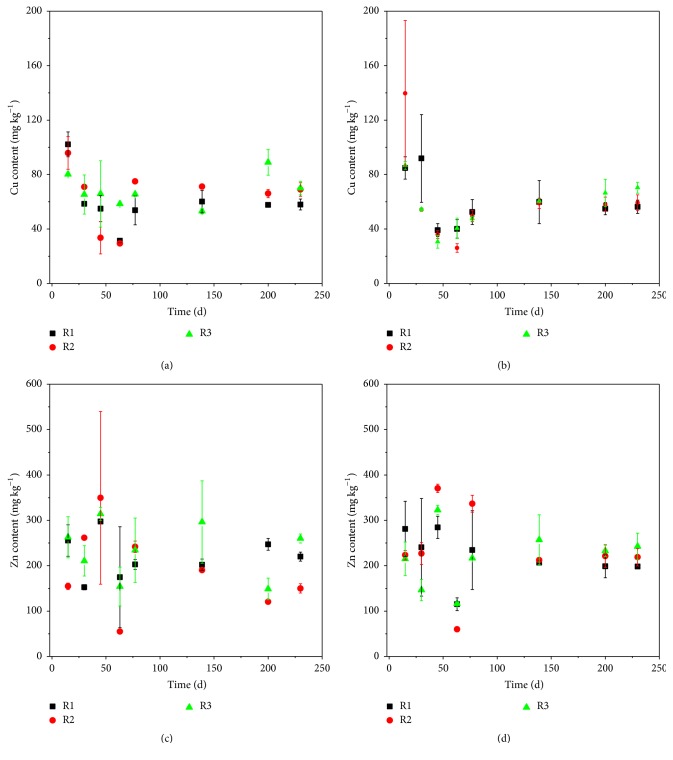
Cu and Zn contents of upper-MSW layer and sub-MSW layer of the simulated landfills. (a) Cu contents of the upper-MSW layer; (b) Cu contents of the sub-MSW layer; (c) Zn contents of the upper-MSW layer; (d) Zn contents of the sub-MSW layer.

The variation of Cu content in MSW of the three simulated landfills is shown in Figures 6(a) and 6(b). The content of Cu experiences a fast reduction and a slow rise during the study period. This result is consistent with the previous research [28]. Due to the heterogeneity of MSW, the content of Cu in MSW is somewhat erratic. No significant difference is observed among the Cu contents of upper-MSW layer of R1, R2, and R3 during the study period. For sub-MSW layer, no significant difference is observed before day 139. After day 139, the average Cu contents of sub-MSW layer follow the sequence of R3 > R2 > R1, which is consistent with the mass proportion of the MSWI bottom ash layer in the simulated landfills. Cu contents of R3 sub-MSW layer are significantly higher than that of R1 (*p* < 0.05, shown in Table S5). During the running of the simulated landfill, the released Cu from the upper-MSW layer and MSWI bottom ash layer was immobilized by the sub-MSW layer with the vertical flow of the leachate. As the contents of Cu follow the sequence of R3 > R2 > R1, the Cu content of sub-MSW layer follows the same sequence. This result suggests that the MSWI bottom ash layer can increase the Cu content of sub-MSW layer. As a matter of fact, the Cu content in MSW of R1 has already exceeded the China Environmental Quality Standard for Soils (GB15618-1995, Grade II for soil pH < 6.5: Cu ≦ 50 mg kg^−1^) [29]. The MSWI bottom ash layer further aggravates the contamination.

#### 3.4.2. Zn

Due to the heterogeneity of MSW, the Zn contents of the upper-MSW layer of R1, R2, and R3 are erratic during the study period (Figure 6(c)). Before day 77, no significant difference is observed between the Zn contents of the sub-MSW layer of R1, R2, and R3 (Figure 6(d)). After day 77, the average Zn contents of sub-MSW layer are 201.21, 217.36, and 244.07 mg kg^−1^ for R1, R2, and R3, respectively, which follows the sequence of R3 > R2 > R1. Zn contents of R3 sub-MSW layer are significantly higher than that of R1 (*p* < 0.05, shown in Table S5).

The MSWI bottom ash layer can increase the Cu and Zn contents of sub-MSW layer of landfill. This result is consistent with the report of Lo et al. [30], which pointed out that MSW had great adsorption capacity for the heavy metals, including Cu and Zn. The adsorption is greatly affected by the pH. A low pH can lessen the binding of metals with MSW [31, 32]. In this respect, the MSWI bottom ash layer can increase the potential threat of landfill. Once there is an acid rain, the heavy metals in MSW will be released into the environment sharply.

## 4. Environmental Implications

The discharge of Zn from the landfill is dependent on the mass proportion of MSWI bottom ash layer. When the mass proportion of MSWI bottom ash layer to MSW layer is 1 : 9, which is not enough to neutralize the acidic condition, the discharge of Zn is enhanced. However, the release of Zn might be mitigated if the mass proportion of MSWI bottom ash layer to MSW layer is 2 : 8, which is able to neutralize the acidic condition. Different from Zn, the discharge of Cu is not greatly affected by the MSWI bottom ash layer, due to the high affinity of Cu to the organic ligands. The organic matter is abundant in all three simulated landfills, resulting in the similar leaching behavior of Cu. With the extension of running time, the released Cu and Zn from MSWI bottom ash layer are immobilized by the sub-MSW layer. Therefore, the Cu and Zn contents of sub-MSW layer are increased. It suggests the MSWI bottom ash layer can increase the potential threat of landfill. Future work will be conducted to explore the optimal addition ratios for the MSWI bottom ash use in the landfill site, as well as the pollution control measures.

## Supplementary Material

The statistical analysis result of Cu concentrations in the leachates is shown in Suppl. Table S1, which suggests that no significant difference is observed among the three leachates in most cases of the study. The calculation of the discharge of Cu is presented in Suppl. Table S2. The statistical analysis result of Cu concentration in the leachates is shown in Suppl. Table S3, which suggests that the Zn concentrations of R2 leachate are significantly higher than that of R1 and the Zn concentrations in R3 leachate are significantly lower or at the same level compared to that of R1. The calculation of the discharge of Zn is presented in Suppl. Table S4. The statistical analysis result of Cu and Zn contents of sub-MSW-layer of R1, R2 and R3 after day 139 is presented in Suppl. Table S5, which shows the MSWI bottom ash layer can increase the Cu and Zn contents of sub-MSW layer of landfill.

## Figures and Tables

**Table 1:** Physicochemical properties and bulk composition of the MSWI bottom ash sample.

Physicochemical properties or element composition	Value/content (mg kg^−1^)
Moisture content (%)	1.61
Bulk density (kg (m^3^)^ −1^)	1277.6
Loss on ignition (LOI) (%)	2.2
pH	11.2
Acid neutralization capacity (ANC_7.5_)	1 H^+^ mequiv g^−1^
Ca	69413 ± 2613
Al	40920 ± 1600
Fe	26008 ± 28
K	15792 ± 167
Zn	1922.0 ± 33.0
Cu	314.60 ± 22.3

**Table 2:** Components and Cu and Zn contents of the MSW [13].

Components	Food waste	Plastic	Paper	Textile	Dust	Ceramic	Metal	Timber	Residue
Weight/weight, %	45.5	8.5	9.5	0.1	5.2	5.8	0.1	0.7	24.5
Cu content (mg kg^−1^)	114.8	175.8	133.3	84.7	148.3	164.0	—	163.7	126.5
Zn content (mg kg^−1^)	1540.5	774.5	929.1	169.4	707.6	761.7	—	834.0	846.3
