# Supplementary material for: Effect of Mass Proportion of Municipal Solid Waste Incinerator Bottom Ash Layer to Municipal Solid Waste Layer on the Cu and Zn Discharge from Landfill
Source: Biomed Res Int. 2016 Dec 1;2016:9687879. doi: 10.1155/2016/9687879 (PMC5156799; doi:10.1155/2016/9687879)
Supplement: Supplementary file 1 — The statistical analysis result of Cu concentrations in the leachates is shown in Suppl. Table S1, which suggests that no significant difference is observed among the three leachates in most cases of the study. The calculation of the discharge of Cu is presented in Suppl. Table S2. The statistical analysis result of Cu concentration in the leachates is shown in Suppl. Table S3, which suggests that the Zn concentrations of R2 leachate are significantly higher than that of R1 and the Zn concentrations in R3 leachate are significantly lower or at the same level compared to that of R1. The calculation of the discharge of Zn is presented in Suppl. Table S4. The statistical analysis result of Cu and Zn contents of sub-MSW-layer of R1, R2 and R3 after day 139 is presented in Suppl. Table S5, which shows the MSWI bottom ash layer can increase the Cu and Zn contents of sub-MSW layer of landfill. [file 9687879.f1.pdf]

Effect of mass proportion of municipal solid waste incinerator bottom ash layer to municipal solid waste layer on the Cu and Zn discharge from landfill

Qingna Kong,[A] Jun Yao,[A,B,D] Zhanhong Qiu,[A] and Dongsheng Shen[C]

[A] Center of marine and ecological environment protection, Taizhou University, Linhai 317000, China

[B] Department of Environmental Engineering, Zhejiang University, Hangzhou 310029, China

[C] Zhejiang Provincial Key Laboratory of Solid Waste Treatment and Recycling, Zhejiang Gongshang University, Hangzhou, 310018, China

[D] Corresponding author. Email: 18806591228@163.com

Table S1. Cu concentrations in leachate of R1, R2 and R3 (mg L^{-1}).

Time	R1	R2	R3
1	3.14±0.06 [a]	3.13±0.09 [a]	3.06±0.03 [a]
7	1.01±0.01 [a]	1.09±0.01 [a]	1.06±0.14 [a]
15	1.78±0.00 [b]	1.16±0.04 [a,b]	0.78±0.55 [a]
22	0.91±0.22 [a]	0.92±0.03 [a]	0.87±0.08 [a]
27	0.70±0.07 [a]	0.62±0.02 [a]	0.59±0.04 [a]
37	0.52±0.07 [a]	0.53±0.03 [a]	0.48±0.03 [a]
44	0.58±0.03 [a]	0.70±0.14 [a]	0.60±0.11 [a]
51	0.78±0.04 [a]	0.80±0.08 [a]	0.80±0.01 [a]
59	0.42±0.03 [a]	0.44±0.02 [a]	0.51±0.04 [b]
65	0.42±0.04 [a]	0.39±0.01 [a]	0.38±0.01 [a]
72	0.05±0.01 [a]	0.09±0.03 [a]	0.06±0.04 [a]
79	0.01±0.01 [a]	0.03±0.02 [a]	0.02±0.02 [a]
88	0.06±0.04 [a]	0.04±0.01 [a]	0.03±0.03 [a]
99	0.17±0.01 [a]	0.33±0.10 [b]	0.24±0.03 [b]
110	0.17±0.01 [a]	0.16±0.01 [a]	0.16±0.03 [a]
119	0.17±0.01 [a]	0.16±0.02 [a]	0.16±0.03 [a]
129	0.21±0.03 [a]	0.24±0.05 [a]	0.17±0.03 [a]
144	0.52±0.01 [a]	0.65±0.06 [b]	0.53±0.02 [a]
159	0.54±0.10 [a]	0.56±0.01 [a]	0.73±0.34 [a]
183	0.51±0.01 [a]	0.49±0.01 [a]	0.42±0.10 [a]
200	0.77±0.01 [b]	0.66±0.06 [a]	0.64±0.01 [a]
230	0.69±0.03 [a]	0.79±0.02 [b]	0.71±0.02 [a]

a, b indicate the independent variables which were significant at $P < 0.05$ level.

Table S2. Calculation of Cu discharge from R1, R2 and R3 (mg)

Time	R1	R2	R3
1	10.1422	10.0786	9.2412
7	0.2727	0.3052	0.23638
15	0.623	0.348	0.8346
22	0.121	0.0544	0.06786
27	0.084	0.0589	0.3704
37	0.0572	0.0477	0.1648
44	0.08294	0.0595	0.702
51	0.0897	0.0832	0.128
59	0.0672	0.05368	0.0918
65	0.063	0.0468	0.0589
72	0.0459	0.01	0.0078
79	0.0105	0.0366	0.002
88	0.057	0.0508	0.00285
99	0.0595	0.1056	0.1944
110	0.0629	0.0592	0.0448
119	0.0578	0.0512	0.0448
129	0.0504	0.0816	0.0459
144	0.13	0.234	0.1484
159	0.1728	0.1512	0.2263
183	0.1173	0.1323	0.105
200	0.1848	0.1122	0.16
230	0.138	0.1738	0.11715
Total	12.68984	12.33448	12.99534

Table S3. Zn concentrations in leachate of R1, R2 and R3 (mg L^{-1}).

Time	R1	R2	R3
1	17.06±0.74 ^{a}	19.41±0.00 ^{b}	23.55±0.26 ^{c}
7	14.78±0.13 ^{a}	15.99±1.42 ^{a}	15.21±0.24 ^{a}
15	13.71±0.53 ^{a}	14.19±0.32 ^{a}	13.61±0.27 ^{a}
22	5.97±0.07 ^{a}	6.92±0.51 ^{b}	6.48±0.64 ^{b}
27	7.24±0.50 ^{a}	6.68±0.39 ^{a}	7.95±1.29 ^{a}
37	12.13±0.06 ^{b}	14.13±0.05 ^{c}	8.24±0.58 ^{a}
51	8.48±2.97 ^{a,b}	7.28±0.16 ^{b}	5.50±0.43 ^{a}
59	9.42±0.13 ^{b}	11.22±0.30 ^{c}	6.75±0.19 ^{a}
65	7.75±0.27 ^{b}	7.51±0.94 ^{a,b}	7.03±0.26 ^{a}
72	10.77±0.13 ^{a}	12.34±0.20 ^{c}	11.77±0.13 ^{b}
79	14.94±0.66 ^{b}	17.20±0.09 ^{c}	13.54±0.34 ^{a}
88	9.16±0.94 ^{a}	9.83±0.19 ^{a}	12.43±0.94 ^{b}
110	7.88±0.47 ^{b}	7.35±0.77 ^{a}	7.79±2.53 ^{b}
119	6.80±0.28 ^{c}	5.73±0.03 ^{a}	6.25±0.11 ^{b}
129	6.37±0.02 ^{b}	6.20±0.60 ^{b}	3.75±0.55 ^{a}
144	3.44±0.47 ^{a}	4.77±0.01 ^{b}	3.69±0.92 ^{a}

159	5.31±0.75 [a]	8.94±1.76 [b]	5.16±0.14 [a]
183	6.77±0.44 [a]	11.26±1.01 [c]	9.04±0.76 [b]
200	7.71±1.30 [a]	10.16±1.28 [b]	6.84±0.13 [a]
230	4.85±0.41 [b]	7.06±0.80 [c]	3.92±0.07 [a]
275	4.81±0.31 [b]	7.01±0.99 [c]	3.91±0.31 [a]

a, b, c indicate the independent variables which were significant at $P < 0.05$ level.

Table S4. Calculation of Zn discharge from R1, R2 and R3 (mg)

Time	R1	R2	R3
1	55.1038	62.5002	71.121
7	3.9906	4.4772	3.39183
15	4.7985	4.257	14.5627
22	1.3134	0.91344	0.50544
27	0.8688	0.6346	0.59625
37	1.3343	1.2717	1.1124
51	0.9752	0.75712	0.88
59	1.5072	1.36884	1.215
65	1.1625	0.9012	1.08965
72	9.88686	15.1782	1.5301
79	15.687	20.984	1.353
88	8.702	12.4841	1.18085
110	2.9156	2.7195	2.1812
119	2.312	1.8336	1.75
129	1.5288	2.108	1.0125
144	0.86	1.7172	1.0332
159	1.6992	2.4138	1.5996
183	1.5571	3.0402	2.26
200	1.8504	1.7272	1.71
230	0.97	1.5532	0.6468
275	2.96296	2.37639	0.72335
Total	121.9862	145.2167	111.4549

Table S5. Cu and Zn contents of sub-MSW-layer of R1, R2 and R3 after day 139 (mg kg^{-1}).

Time	Cu			Zn		
	R1	R2	R3	R1	R2	R3
139	59.80±15.80 [a]	58.82±3.79 [a]	60.53±0.52 [a]	206.68±4.46 [a]	212.17±1.78 [b]	257.07±55.01 [b]
200	54.91±4.44 [a]	58.25±5.18 [a,b]	66.42±10.02 [b]	198.62±25.08 [a]	221.25±25.18 [a,b]	232.58±12.46 [b]
230	56.32±4.88 [a]	59.33±5.78 [a]	70.34±3.45 [b]	198.34±4.77 [a]	218.67±19.23 [b]	242.55±29.30 [b]

a, b indicate the independent variables which were significant at $P < 0.05$ level.
